# Multiple Myeloma: Risk Stratification and Toxicity Management

**Published:** 2018-04-01

**Authors:** Tiffany Richards, Hans Lee

**Affiliations:** The University of Texas MD Anderson Cancer Center, Houston, Texas

## Abstract

The treatment landscape for multiple myeloma is rapidly changing. At JADPRO Live 2017, presenters brought advanced practitioners up to date on the newest approaches to the management of this disease, as well as what to be aware of when treating common side effects.

Multiple myeloma is a complex malignancy for which the treatment landscape is rapidly changing. Risk stratification is important in determining prognosis and tailoring treatment. At JADPRO Live 2017, Tiffany Richards, PhD, ANP, AOCNP®, and Hans Lee, MD, both from The University of Texas MD Anderson Cancer Center in Houston, brought listeners up to date on the newest approaches to management.

Multiple myeloma is a malignancy of the plasma cells that can evolve over time. Almost all patients are initially diagnosed with monoclonal gammopathy of unknown significance (MGUS). Approximately 1% of MGUS patients per year will develop "smoldering myeloma," and about 10% per year of these patients will eventually develop symptomatic disease, Dr. Richards said.

The International Myeloma Working Group (IMWG) revised the diagnostic criteria for myeloma in 2014, which now incorporates biomarkers along with the standard "CRAB" criteria (hypercalcemia, renal insufficiency, anemia, bone lesions), and guides the initiation of treatment (Rajkumar et al., 2014). In the absence of CRAB criteria, myeloma-defining events are bone marrow clonal plasmacytosis ≥ 60%, free light chain ratio ≥ 100, and focal lesions at least 5 mm in size on magnetic resonance imaging.

For MGUS, most patients can be followed (i.e., observed), with evaluations every 3 to 6 months for the first year, then annually thereafter. Patients with smoldering multiple myeloma can also be followed closely or enrolled in a clinical trial for smoldering disease. Studies are now evaluating whether aggressive treatment early on can affect outcomes.

## RISK STRATIFICATION OF PATIENTS

Once a diagnosis of multiple myeloma has been made, risk stratification is the next step, as discussed by Dr. Lee. "There have been tremendous advancements in the treatment of myeloma over the past 10 to 15 years, mainly due to the approval of new drugs and resulting in a doubling of overall survival," he said. Since 2013, the treatment armamentarium has grown to include carfilzomib, pomalidomide (Pomalyst), panobinostat (Farydak), daratumumab (Darzalex), ixazomib (Ninlaro), and elotuzumab (Empliciti).

The life expectancy of standard-risk myeloma patients is now 10 to 12 years, but this falls to 3 years for patients with high-risk cytogenetics, despite the use of the newest agents. Risk stratification, therefore, is important for prognosis and for identifying candidates for novel treatments and clinical trials. High risk is defined by disease biology (by molecular classifications derived from cytogenetics/fluorescence in situ hybridization [FISH], gene expression profiling, and next-generation sequencing); by phenotype (presence of plasma cell leukemia or extramedullary disease); by disease burden (beta2-microglobulin, albumin, lactate dehydrogenase); and by response to therapy.

A number of chromosomal translocations, deletions, and amplifications have prognostic significance, particularly those involving the IgH heavy chain locus on chromosome number 14 and hyperdiploidy. Abnormalities of note include deletions 17p and 1p and translocations (4;14) and (14;16). Identification of these abnormalities by FISH is a critical part of risk stratification. The risk stratification approach is staging via the myeloma International Staging System (ISS), which was revised in 2015 to incorporate FISH studies. The IMWG has produced a simple table for risk stratification that is useful to clinicians (Table 1).

**Table 1:**
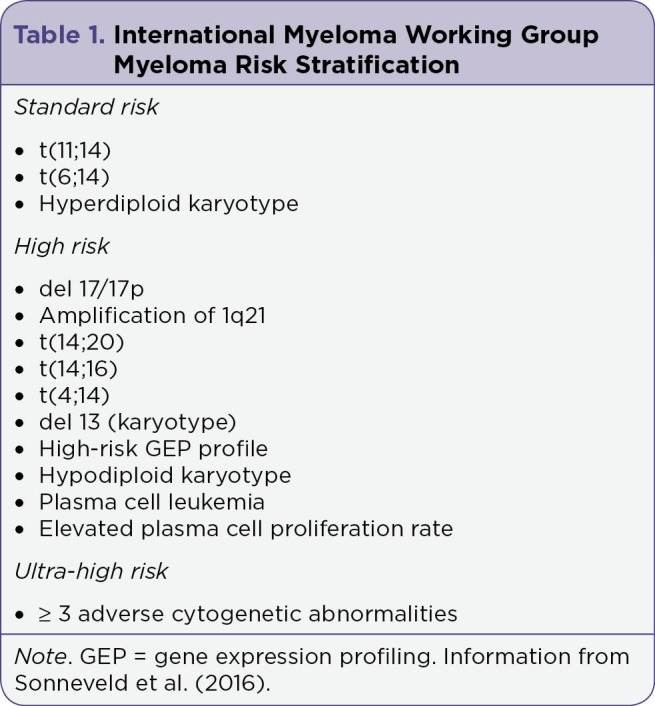
International Myeloma Working Group Myeloma Risk Stratification

## INITIAL TREATMENT SELECTION

With many options available now, how do clinicians select an initial therapy for a newly diagnosed patient? The first question is whether the patient is a candidate for autologous stem cell transplant. The next step is to determine which frontline therapy should be used beforehand, Dr. Richards said. 

For upfront treatment, a proteasome inhibitor has proven critical for good long-term outcomes. In SWOG S0777, the triplet of bortezomib (Velcade)/lenalidomide (Revlimid)/dexamethasone (VRd) proved clearly more effective than the lenalidomide/ dexamethasone (Rd) doublet (Durie et al., 2017). The overall response rates were 81.5% vs. 71.5%; rates of very good partial response or better were 43.5% and 31.8%, respectively; and 15.7% vs. 8.7%, respectively, achieved a complete response. Importantly, in the high-risk subgroup, VRd more than doubled progression-free survival (*p* = .0037), exceeding 34 months. Similarly, in the whole population, overall survival was improved from a median of 64 months with Rd to 75 months with VRd (*p* = .0250).

In other studies, bortezomib/thalidomide/dexamethasone provided better outcomes than the triplet of bortezomib/cyclophosphamide/dexamethasone (CyBorD), one being the French IFM 2013-04 trial (Moreau et al., 2016). Another European study compared bortezomib/thalidomide/dexamethasone (VTD), which is assumed to be similar to VRd, to CyBorD, and also showed a doubling in rates of very good partial response or better with bortezomib on board. Of note, in high-risk subgroups, multiple randomized trials have shown that bortezomib-containing regimens improve survival (Sonneveld et al., 2016).

Carfilzomib (Kyprolis) is the newer proteasome inhibitor. Two important phase II studies showed that, with carfilzomib combined with Rd, complete or near complete responses were achieved by 52% and 62% of patients, respectively (Jakubowiak et al., 2012; Korde et al., 2015). In one study, two-thirds of patients were negative for minimal residual disease, and in both studies 92% of patients were progression-free at 18 or 24 months. All patients with stage 1 or 2 disease responded to the treatment, as did 93% with stage 3 disease and 94% of patients with "unfavorable" features. "We see very high response rates, as well significant progression-free survivals, in patients receiving carfilzomib," Dr. Richards noted.

Ongoing trials are comparing these effective triplets, studying VRd in combination with monoclonal antibodies, and asking whether VRd can delay the need for transplant, she added.

In patients not eligible for transplant, the use of Rd as maintenance after frontline therapy, given either for 18 months or continuously, proved effective in the FIRST trial (Benboubker et al., 2014; Facon et al., 2013). Progression-free survival was highest, at 25.2 months, among the patients continuously treated with Rd.

"So, how do we decide what we’re going to treat our patients with? If the patient is transplant eligible we recommend a triplet regimen. The current standard of care is VRd, but you may want to consider carfilzomib/lenalidomide/dexamethasone (CRd) in patients with high-risk disease. You definitely do not want to give melphalan, which can impact your collection of stem cells," Dr. Richards advised. "For those who are transplant ineligible, based on the patient’s frailty and comorbidities you could use a doublet, such as Rd, or you could consider triplet therapy, such as VRd, with maintenance given after initial therapy. You would also want to consider lower doses."

## AUTOLOGOUS STEM CELL TRANSPLANT

After completion of frontline therapy, is stem cell transplant still needed? With the emergence of many effective novel therapies, myeloma specialists have begun to debate this concept, according to Dr. Lee. The most recent and best study addressing this question is the Dana Farber Cancer Institute/IFM 2009 trial, in which patients received three cycles of VRd with stem cell collection, with or without transplant, plus maintenance lenalidomide (Attal et al., 2017). Results have been reported only for the French IFM 2009 component, showing that VRd plus transplantation significantly prolonged progression-free survival over VRd alone (50 months vs. 26 months; hazard ratio [HR], 0.65; *p* < .001). Overall survival at 4 years, however, did not differ significantly, "suggesting that patients who deferred on their initial stem cell transplant could be salvaged by stem cell transplant later on in their disease course," he said.

Whether transplant should be performed upfront or can be delayed is still an "evolving question," he continued. Relevant issues are the role of indefinite maintenance with lenalidomide and its effect on long-term outcomes (which should be answered by the Dana Farber component of the trial) and the role of minimal residual disease negativity as a clinically relevant endpoint in deciding upon upfront vs. delayed transplant.

Lenalidomide maintenance has become an accepted component of care, based on CALGB 100104 in which maintenance improved progression-free survival by 19 months and improved 3-year overall survival as well (McCarthy et al., 2012). Likewise, the IFM 2005-02 study also showed that lenalidomide maintenance post-transplant extended progression-free survival, but not survival (Attal et al., 2012). Lenalidomide maintenance therapy also benefited patients with high-risk cytogenetic abnormalities in these studies.

While maintenance with lenalidomide alone is probably sufficient for standard-risk patients, there is a trend toward combining an immunomodulatory drug (IMiD) and proteasome inhibitor for maintenance in high-risk patients. In a phase II study from Emory University, the VRd triplet as maintenance after transplant showed promise for patients with high-risk cytogenetics (Nooka et al., 2014).

## TREATMENT AT RELAPSE

"For relapsed/refractory myeloma, there are many different options now. It’s good news, but it can also be quite overwhelming to patients," Dr. Richards commented.

In selecting a treatment option, clinicians need to determine the goal of treatment, and then look at the patient’s previous treatment and response to it, duration of remission, previous toxicities, other disease-related factors, and comorbidities. If an appropriate clinical trial is available, this should also be considered.

If it is an asymptomatic biochemical relapse in a standard-risk patient, treatment options are more flexible. Patients may do well on observation only, or on a doublet or an all-oral regimen; patients with high-risk disease, on the other hand, warrant prompt intervention. "When those patients start to relapse, they take off very quickly," she noted. For patients displaying an aggressive clinical relapse, a daratumumab- or a carfilzomib-based regimen can be considered. Treatment should always be tailored in a way that balances efficacy with quality of life and adheres to the patient’s goals, Dr. Richards emphasized.

The National Comprehensive Cancer Network has listed a number of triplets as its preferred regimens for relapsed disease, and one doublet, carfilzomib/dexamethasone. In the ENDEAVOR trial, carfilzomib/dexamethasone was superior to bortezomib/dexamethasone, including in high-risk patients, who achieved a 15.5% complete response rate vs. 4.4% with bortezomib/dexamethasone (Dimopoulos et al., 2016). But when carfilzomib was combined with Rd (CRd), the ASPIRE trial documented a very high median progression-free survival of 26.3 months, vs. Rd alone (HR, 0.69; *p* = .0001; Stewart et al., 2015). The oral proteasome inhibitor ixazomib, combined with lenalidomide/dexamethasone, was also impressive in a phase III trial in which median progression-free survival was 20.6 months, vs. 14.7 months with Rd alone (HR, 0.74; *p* = .01; Moreau et al., 2016). In high-risk patients, the triplet was associated with a 46% reduction in risk of progression.

Carfilzomib/pomalidomide/dexamethasone has also been shown to improve outcomes in high-risk patients. In one study (Shah et al., 2015), MM-003, an 80% response rate was seen among patients with del(17p), even higher than the 50% response rate in the overall population. Regimens involving carfilzomib and ixazomib, therefore, are good alternatives for relapsed high-risk patients, Dr. Richards said.

## MONOCLONAL ANTIBODIES FOR RELAPSE

For relapsed/refractory multiple myeloma, the monoclonal antibodies—daratumumab and elotuzumab—represent a significant advancement in treatment, Dr. Lee indicated. Elotuzumab was FDA-approved in combination with lenalidomide and dexamethasone based on the ELOQUENT-2 trial, in which response rates were 79% for patients in the elotuzumab arm, vs. 66% with Rd alone, and median progression-free survival was 19.4 vs. 14.9 months (HR, 0.70; *p* < .001; Lonial et al., 2015).

Daratumumab became approved as a single agent after producing a 36% response rate in heavily pretreated patients (Lokhorst et al., 2015). Following this, in the CASTOR trial the regimen of daratumumab/bortezomib/dexamethasone was associated with an 83% response rate, vs. 63% for Vd alone, and a median progression-free survival that was not reached, vs. 7.2 months with Vd (HR, 0.39; *p* < .001; Palumbo et al., 2016). In combination with Rd, in the POLLUX study, response rates reached 93%, median progression-free survival was not reached, and 1-year progression-free survival was 83.2% (Dimopoulous et al., 2016).

With the monoclonal antibodies, infusion reactions are common, seen in about 10% of patients receiving elotuzumab but in up to 50% getting their first dose of daratumumab (diminishing to< 5% thereafter). Steps can be taken to greatly mitigate this risk (Table 2). Premedication is important, as is monitoring for infusion-related signs. Pulmonary function should be checked before initiating daratumumab.

**Table 2:**
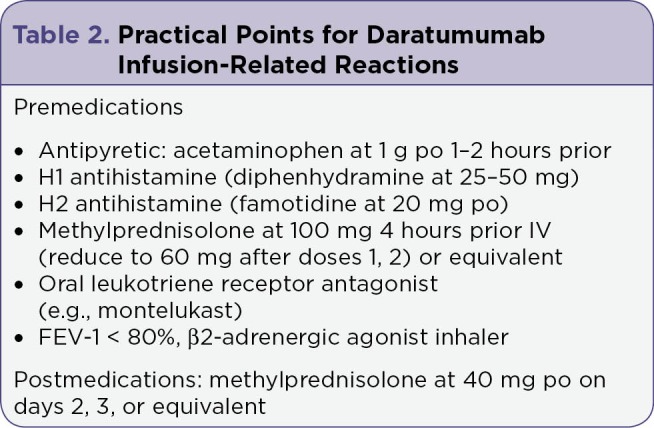
Practical Points for Daratumumab Infusion-Related Reactions

Since daratumumab can also interfere with red blood cell (RBC) compatibility testing, local blood banks should be alerted to any patients initiated on this drug, and patients should have RBC phenotyping or genotyping prior to starting the drug. Patients should be closely monitored for reactions when receiving an RBC transfusion. There are other special considerations with lab tests (especially serum protein electrophoresis, immunofixation, and flow cytometry) in patients receiving these two monoclonal antibodies, Dr. Lee indicated.

## SIDE EFFECTS OF STANDARD TREATMENTS

These effective agents can be well tolerated, although there are some common side effects, as described by Dr. Richards (Table 3).

**Table 3:**
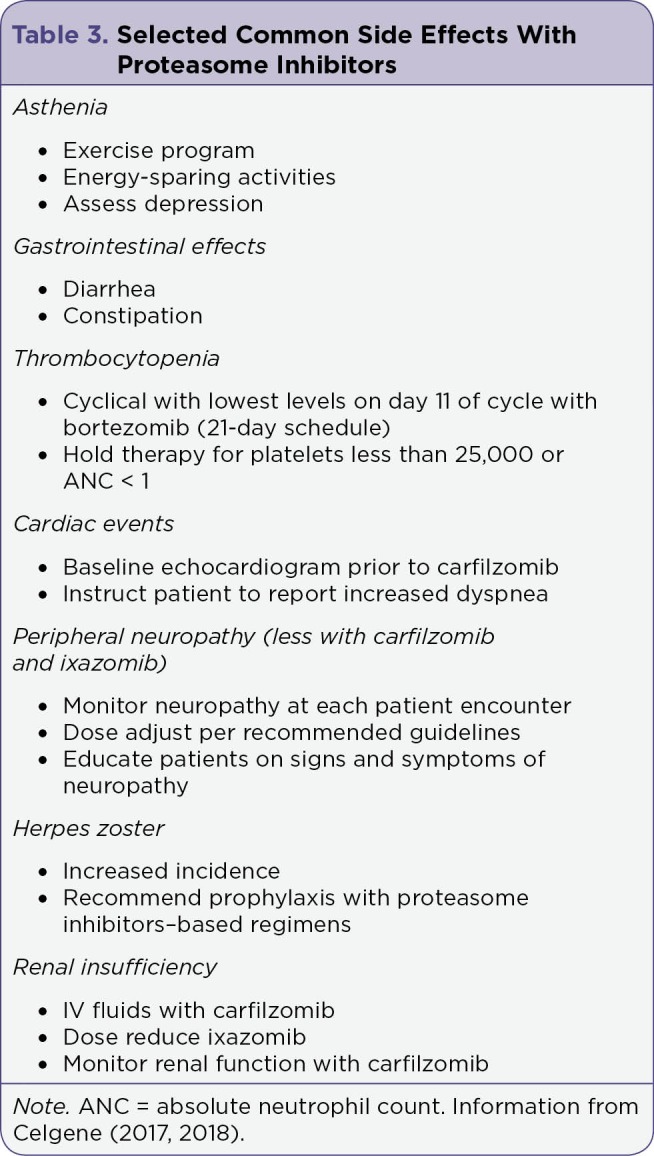
Selected Common Side Effects With Proteasome Inhibitors

Peripheral neuropathy occurs in approximately 75% percent of previously treated patients. Advanced practitioners should be proactive about this toxicity and monitor for it at each visit. Better than using "descriptors," she said, is telling patients " ’Take note of what your hands and feet feel like right now and if that changes, then call so that I can assess you and determine if this is something that we need to be concerned about.’ But don’t just take the patient’s word for it; we know they hide their symptoms. Do your physical exam and look at how they’re walking, test their muscle strength, and make sure they have fine motor movement," she advised.

The development of neuropathy signals the need for immediate dose adjustment. "We tend to think about grade 1 as not a big deal, but it’s actually like having your leg asleep all the time. If we dose adjust early, then we can prevent that from worsening," she pointed out. Neuropathy risk is less with subcutaneous bortezomib, carfilzomib, and ixazomib, than with intravenous bortezomib.

Patients with neuropathy should be assessed for vitamin B12, B6, and folate deficiency (which can cause it). Gabapentin, pregabalin, duloxetine, acupuncture, physical therapy, and aquatherapy may be helpful, and there is some evidence that glutamine is preventive, she added.

Also seen with proteasome inhibitors is an increased risk of herpes zoster. Prophylaxis with valacyclovir or acyclovir is critical, and patients must be instructed to stay on these antivirals. Patients with renal insufficiency should be given intravenous fluids prior to dosing with carfilzomib, but watch for fluid overload; the recommended amount is 250 mL. When creatinine clearance is < 30 mL/min, ixazomib should be dose-reduced. Renal function should also be monitored in patients taking carfilzomib, she said.

With IMiDs, fatigue, gastrointestinal problems, thrombocytopenia, neutropenia, peripheral neuropathy, renal dysfunction, peripheral neuropathy, and rash can be issues. Rash, which occurs in up to 20% of patients on IMiDs, can be mitigated with the combination of cetirizine, ranitidine, and L-lysine, after stopping the lenalidomide. When rash resolves, many patients can be restarted on the same initial dose, without recurrence.

Risk for thromboembolic events is high in all cancer patients; clinicians should look for other risk factors and anticoagulate with a daily aspirin, full-dose warfarin, or low-molecular-weight heparin. Since risk factors for thromboembolism can change over time, reassessment is important. Patients should know the symptoms and be encouraged to be mobile.

## INFECTION RISK

For a number of reasons, myeloma patients have a 7-fold increased risk for bacterial infections and a 10-fold risk for viral infections. "Be aware of this risk," Dr. Richards emphasized. In patients with frequent infections, risk can sometimes be reduced by monthly intravenous immunoglobulin treatment and by antibiotic prophylaxis. Patients receiving a proteasome inhibitor or a monoclonal antibody should also be on antiviral prophylaxis. Patients should receive appropriate vaccines (including flu vaccine and pneumonia vaccines), but not live vaccines.

Patients should understand the importance of proper handwashing and should avoid people who are ill (or if unavoidable, wear a mask). Patients traveling outside of the country should be immunized appropriately.

## ADHERENCE TO IV AND ORAL MEDICATIONS

Patients must understand the importance of staying on their medications, and of recognizing and reporting adverse events so that doses can be modified. "You want to reinforce the rationale for the ongoing treatment plan. It’s important that patients understand that if we can keep them on therapy, even at a reduced dose, that’s better than them not taking their medication or skipping doses," Dr. Richards said.

Medication calendars, especially for patients on all-oral regimens, can be very helpful to patients and caregivers. She also told listeners to watch for treatment fatigue in their patients, and be willing to discuss treatment breaks when the disease is stable. Practitioners should also be aware that financial issues can be barriers to adherence, as can depression.

## TAILORING TREATMENT

With multiple drugs available, treatment can to some degree be tailored to the patient, Dr. Richards continued. For example, for a patient with preexisting neuropathy, a carfilzomib-based regimen may be preferred over bortezomib. For a patient with cardiomyopathy, a bortezomib-based regimen may be safer than carfilzomib. For a patient with renal failure, one might consider CyBorD to induce a rapid response. For patients with diabetes, the engagement of the primary care provider or endocrinologist is important, since steroids can cause diabetes. Patients with a history of bleeding should probably avoid an IMiD plus dexamethasone in favor of a proteasome inhibitor plus alkylating agent, which will avoid the need for thromboprophylaxis.
